# Network Analysis on Multi-Domain Loneliness Factors Among Three Aging Groups with Kinship Vulnerability

**DOI:** 10.1093/geroni/igaf122.3497

**Published:** 2025-12-31

**Authors:** Jianan Li

**Affiliations:** University of California, Los Angeles, Los Angeles, California, United States

## Abstract

Close kin (i.e., a partner and children) has been identified as important supporting systems against loneliness in later life, with those with kinship vulnerability—lacking traditional family structures—facing heightened risk. While multi-dimensional factors contributing to loneliness have been studied, their associations across different kinship vulnerability groups remain unexplored. Using the 2016 and 2018 Health and Retirement Study, this study employed network analysis to construct partial correlation networks with 22 factors across six domains (demographics, socioeconomic status, physical, psychological, cognitive, and social well-being) among adults over 50 with kinship vulnerability across three groups: having a partner but no children (“Partner-only,” N = 331), having children but no partner (“Children-only,” N = 2635), and having no partner and no children (“kinless,” N = 487). Centrality measures (betweenness, closeness, strength) showed that psychological well-being factors (depression, hopefulness) were central for Partner-only and Kinless groups, while physical health and demographic factors were prominent for the Children-only group. Ego network analyses revealed that social well-being factors had the strongest direct connections with loneliness: friend support was positively associated with loneliness in Partner-only adults (r=.296), while friend strain was the strongest predictor in Children-only (r=.270) and Kinless groups (r=.255). PageRank analysis identified self-reported health as the most influential bridge across all three networks, especially for Partner-only and Kinless groups. These findings demonstrate distinct loneliness mechanisms across kinship vulnerability groups, informing tailored interventions targeting central factors, factors with the strongest direct and indirect effects, and critical bridges between factors to effectively address loneliness in older adults based on their specific kinship vulnerability.

